# Microclimate and Dry Years Interfere With Landscape Structure Effects on Intraspecific Trait Variation

**DOI:** 10.1002/ece3.71417

**Published:** 2025-05-13

**Authors:** Santiago Ordonez, Balázs Deák, Orsolya Valkó, Vivien Szász, Krisztina Verbényiné Neumann, Anna Mária Csergő

**Affiliations:** ^1^ Institute of Horticulture Hungarian University of Agriculture and Life Sciences Budapest Hungary; ^2^ ‘Lendület’ Seed Ecology Research Group, Institute of Ecology and Botany HUN‐REN Centre for Ecological Research Vácrátót Hungary; ^3^ Department of Nature Conservation & Landscape Management Hungarian University of Agriculture and Life Sciences Gödöllő Hungary; ^4^ Department of Botany Hungarian University of Agriculture and Life Sciences Budapest Hungary

**Keywords:** climate change, functional biogeography, geographic isolation, great Hungarian plain, habitat area, habitat connectivity, heat load, *Salvia nemorosa*

## Abstract

Predicting how changes in climate and land use jointly impact populations is a pressing task in ecology. Microclimate plays a key role in species' local persistence by modulating regional weather effects. However, empirical evidence remains limited regarding the relative effects of landscape structure and microclimate conditions on intraspecific trait variation. Using a spatially and temporally replicated demographic dataset, we tested the relative effects of landscape structure (area and connectivity of remnant habitat fragments), microclimate (heat load), and annual weather conditions (study year) on intraspecific variation in plant traits. We also investigated whether local heat load modulated the weather effects on the traits studied. We performed repeated measurements of stem height, leaf area, number of stems, main inflorescence length, and number of primary side inflorescences of 569 permanently marked individuals of the grassland specialist *Salvia nemorosa* L. We sampled 13 populations encompassing microhabitats exposed to different heat load levels over three consecutive years. Mature individuals had fewer stems in isolated and taller stems in small habitat fragments. High levels of heat load and dry years affected negatively all measured traits, and the negative effects of exposure to high heat load were generally exacerbated in dry years. Our results suggest that exposure to strong environmental stressors may complicate the detection of the real effect of human impact on plant populations. Effective landscape planning for the conservation of dry grassland species should prioritize not only improved habitat connectivity but also the maintenance of habitats with heterogeneous microclimates capable of buffering weather extremes.

## Graphical abstract

We tested the relative effect of landscape structure, microclimate and between‐year fluctuation in weather conditions on intraspecific plant trait variation in 13 populations of a dry grassland specialist plant. We found that a strong heat load gradient modulated by temporal fluctuations in weather conditions were dominant drivers of trait variation, and for some traits these effects were superimposed on constraints due to land use changes. Our results suggest that intraspecific trait variation along local heat stress gradients may interfere with plant responses to landscape reconfiguration, complicating the detection of the real effect of human impact on plant populations.

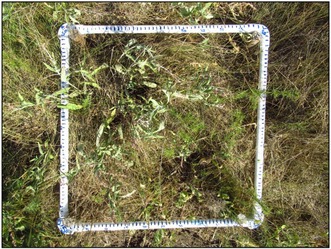

## 1 Introduction

Changes in land use have severely rearranged the structure of natural landscapes, causing habitat fragmentation and loss of habitat amount and connectivity (Plieninger et al. 2016). With accelerated climate change, ecologists are pressed to understand how populations affected by the modified landscape structure cope with altered weather patterns (Maclean and Early 2023; Plieninger et al. 2016). Microclimate may modulate the regional weather effects, thus playing a key role in the persistence of plant populations in remnant habitat patches. The combination of ecological pressures acting at different spatial and temporal scales may enhance plant trait variation and amplify or alleviate the risk of local extinction (McKeon et al. 2023). Accurate forecasts of population persistence are currently impeded by lack of empirical evidence for relative effects of landscape structure, macro‐ and microclimate on intraspecific trait variation (Griffen and Drake 2008; Opedal et al. 2015).

The modified landscape structure may affect plant populations in predictable ways (Fahrig 2017; Hanski and Ovaskainen 2003; Ibáñez et al. 2014; Zambrano et al. 2019). For example, habitat fragmentation can negatively impact species' reproductive success and dispersal by reducing the diversity of pollinators and seed dispersers, which exerts differential pressures on plants with different life histories (Girão et al. 2007; Lopes et al. 2009; May et al. 2019). Trait shifts attributed to habitat fragmentation are often due to isolation effects, because connectivity loss can be an aspect of this process, and fragmentation effects are higher in the most isolated and smallest habitat patches (Fahrig 2003, 2017; Haddad et al. 2015; Hanski and Ovaskainen 2003; Ottaviani et al. 2020). Negative effects specifically attributed to loss of connectivity include hindered pollen and seed dispersal that may cause genetic deterioration and drift (Deák et al. 2018; Ibáñez et al. 2014; Tewksbury et al. 2002; Uroy et al. 2019), and consequently lowered survival chances of species that invest in pollen and seed dispersal compared to species that invest more in other fitness‐related traits (Jacquemyn et al. 2012; Matesanz et al. 2017; Deák, Bede, et al. 2021; Deák, Rádai, et al. 2021). Local populations may respond to isolation via plastic or adaptive trait shifts, e.g., an increased pappus width enabled increased seed dispersal distance (Dener et al. 2021), selection for increased autogamy improved female reproductive fitness in plants with pollination limitation (Xiao et al. 2016), and seed numbers lowered due to reduced chances of dispersal (Rossetto and Kooyman 2005). Thus, geographic isolation triggers spatial variation in traits between populations globally, yet its strength may vary across biological systems (Csergő et al. 2024).

The reduction of the suitable habitat area for certain species is another common attribute of fragmented landscapes, and it can negatively affect a large array of plant traits related to dispersal, establishment, and persistence (Haddad et al. 2015; Zambrano et al. 2019). Due to small population size and consequently lowered genetic diversity and increased inbreeding, small habitat area may decrease reproduction and increase local extinction rates (Zambrano et al. 2019). A low amount of area available for colonization along with modified interspecific relationships (e.g., increased competition and herbivore pressure) may also cause failure to establish and grow, e.g., lower seed number and quality, shorter and fewer stems, shorter inflorescences (Lowe et al. 2005; Weber and Kolb 2011; Zambrano et al. 2019). As with isolation, local populations may respond to constraints from small habitat area and associated processes with plastic or adaptive trait shifts. Increased competition in small habitat fragments could cause trait shifts towards more acquisitive strategies, e.g., fewer and taller stems and larger leaf areas, and limited reproduction (Ibáñez et al. 2014). Several evolutionary trait shifts were related to increased postdispersal competitive advantages and likelihood of establishment in small habitats: increased resprouting ability, seed weight, and seed size (Burns 2016; Kavanagh and Burns 2014). Thus, small habitat area exerts differential pressures on species with different life histories, underlying spatial variation in trait diversity (Herceg‐Szórádi et al. 2023; Ibáñez et al. 2014).

In addition to spatial constraints due to reconfigured habitats, plant populations in remnant habitat fragments face limitations due to the warming climate and restricted availability of suitable microhabitats for establishment or growth (McKeon et al. 2023; Riba et al. 2002). Plant traits related to light and water use are the most sensitive to higher air temperature, light availability, and drought stress specific to remnant habitat fragments (Magnago et al. 2014). Rising temperatures and associated drought are therefore expected to trigger exacerbated responses of these traits in fragmented habitats. Expected effects include decreased specific leaf area (SLA) and plant height for traits involved in persistence, and decreased flower or inflorescence number, but increased inflorescence size for traits involved in establishment (Descamps et al. 2021; Niinemets 2001; Wright et al. 2004). However, the amount of heat that individuals receive through solar radiation can vary in areas with a heterogeneous topography. For example, southern and southwestern slope aspects in the Northern hemisphere receive solar radiation for longer periods and with greater intensity compared to northern and northeastern slope aspects, creating a warmer and drier environment (He et al. 2017). As a result, microclimate, known to directly influence the ecophysiology of individuals, can modulate the effect of macroclimate conditions on plant traits, and ultimately, the availability of suitable microclimates will drive the overall performance of local populations (De Frenne et al. 2013; Deák et al. 2024; Fekete et al. 2023; Kemppinen et al. 2024). For example, higher variation in leaf size and increased leaf mass per area was reported on plant individuals growing on south‐facing slopes compared to north‐facing slopes, suggesting diversity in plant water use and leaf balance energy depending on exposure to local conditions (Ackerly et al. 2002; Li et al. 2021).

Furthermore, despite major advances in functional landscape ecology, trait‐based models of habitat fragmentation syndromes are often limited to snapshot data records. These approaches lack the ability to correctly predict future chances of population persistence in fragmented landscapes under climate change, for which repeated measurements over several years, in populations subject to different macro‐ and microclimate conditions, are necessary (Buckley and Puy 2022; Christiansen et al. 2024; Holt et al. 2022). Repeated measurements were key to our understanding of the effects of water availability on hydraulic and leaf traits (Anderegg 2015) or environmental variation effects on fruit morphology (Sato et al. 2000; Yamada et al. 1993). However, due to inherent data collection difficulties, studies of intraspecific trait variation in fragmented landscapes replicated both spatially and temporally are not common (Nicolè et al. 2011; Valdés et al. 2014).

We addressed this knowledge gap, and we examined the relative effect of habitat area and connectivity, local heat load, and between‐year weather variation on intraspecific trait variation in fragmented *Salvia nemorosa* L. populations over three consecutive years. We examined whether vegetative traits involved in the process of plant persistence (number of stems, stem height, leaf area) and generative traits involved in the process of plant establishment (main inflorescence length and number of side inflorescences) show effects normally expected due to limiting factors, or, on the contrary, they show plastic or adaptive shifts expected in response to those limitations (Table 1). We expected that (i) in isolated and small habitat fragments, plants potentially negatively impacted by genetic deterioration or modified biotic interactions would have limited growth and reproduction, e.g., a lower number of stems and shorter stems. However, we hypothesized that plants might have responded to these limitations to avoid the negative consequences of impeded persistence and establishment. Thus, in isolation, plants could have developed taller stems and longer main inflorescences better visible to pollinators, or more side inflorescences to extend the chances of successful pollination; likewise, in small habitats, plants could have developed taller stems and larger leaf area as a response to the likely increased interspecific competition. We further expected that (ii) in microhabitats exposed to excessive levels of heat load, both the vegetative and generative traits will be constrained compared to habitats with more benign microclimates. However, we expected that the main inflorescence length may have increased under high levels of heat load, to improve the chances of early season reproduction. Finally, we expected that (iii) trait responses to elevated heat load would be exacerbated in years with hot and dry weather.

**TABLE 1:** Table showing the measured *Salvia nemorosa* traits, the plant processes involved, and expected trait responses to constraints by geographic isolation, small area, and heat stress.

Measured trait	Plant process	Isolation (Genetic deterioration and drift due to limited pollen and seed dispersal)	Small area (Genetic deterioration due to small population size, modified biotic interactions)	Heat load (microclimate)	Heat and water stress (hot and dry years)
Number of stems	Persistence	−	− + Increased bud bank (number of stems) to increase the likelihood of individual persistence	−	−
Stem height	Persistence	− + Increased visibility for pollinators	− + Improved light acquisition in response to competition	−	−
Leaf area	Persistence	−	− + Faster resource acquisition in response to competition	−	−
Main inflorescence length	Establishment (early season)	− + Increased visibility for pollinators	−	− + Boosted early season reproduction	− + Boosted early season reproduction
Number of side inflorescences	Establishment (late season)	− + Bet‐hedging, extended chances of pollination	− + Bet‐hedging, extended chances of establishment	−	−

*Note:* The negative sign indicates effects normally expected due to limiting factors, and the positive sign indicates possible trait shifts in response to those limitations.

## 2 Methods

### 2.1 Study Sites

We chose kurgans as a model system. Kurgans are ancient burial mounds built in large numbers during the Late Copper, Bronze, and Iron Ages throughout the steppe biome in Eurasia (Deák 2020). Primarily due to intensive agricultural practices, approximately 57% of pristine European steppe grasslands on Chernozem soils have been destroyed or abandoned (Török et al. 2016). Due to their morphology and cultural significance, many kurgans escaped cultivation, representing one of the last refuges for populations of steppe and dry grassland species in intensively used landscapes (Deák 2020). Being separated from each other and other grasslands by croplands, farmlands, and roads, they represent a good example of habitats with severed connectivity, which may be challenging to overcome for species with modest dispersal abilities such as *S. nemorosa* (Novák and Konvička 2006). Previous studies found kurgans to favor species with large seed mass, tall growth, and autonomous reproduction (Deák, Bede, et al. 2021), and to have lower plant density of specialist species compared to large steppe enclaves (Dembicz et al. 2016).

**FIGURE 1:**
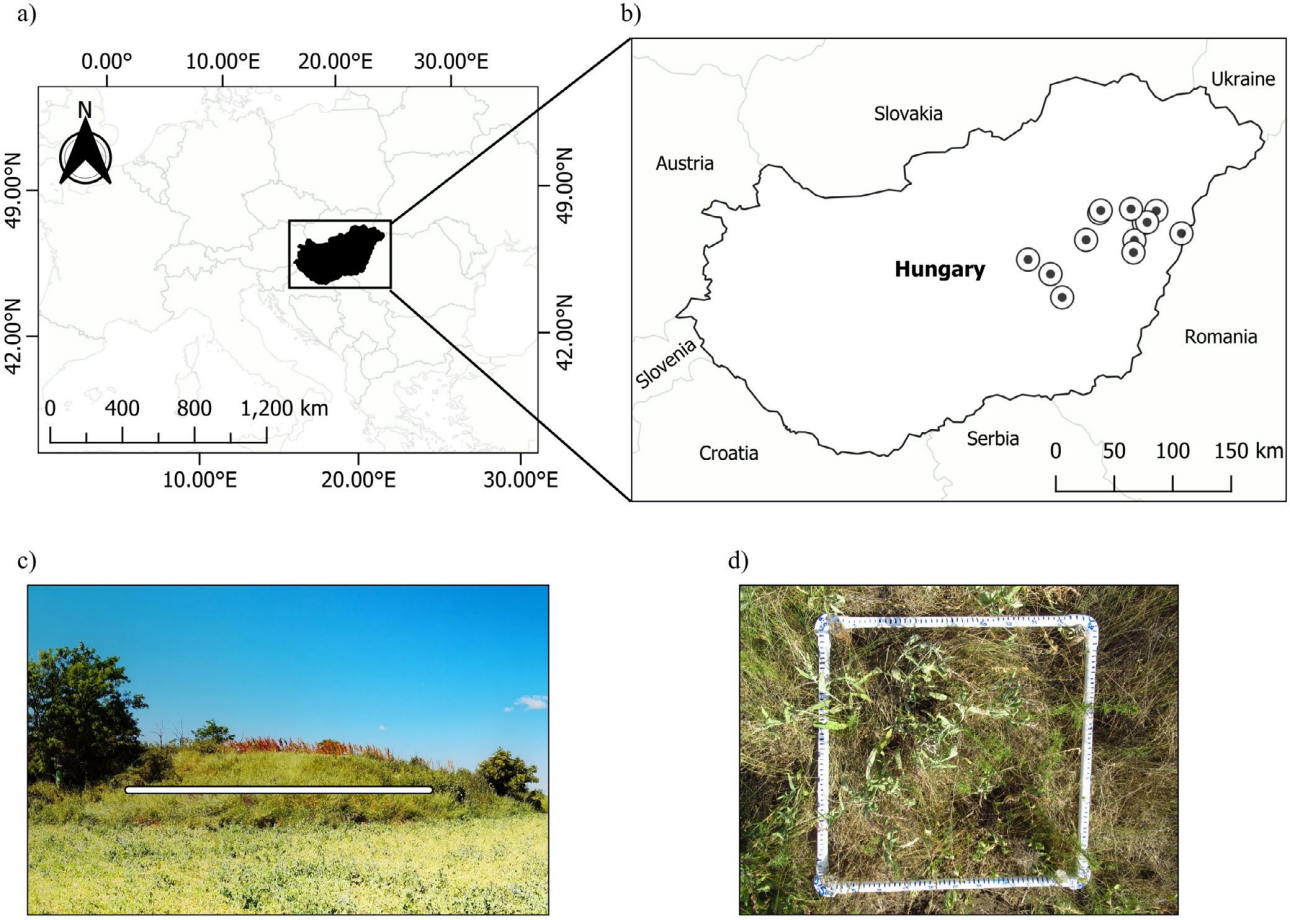
(a) Map showing the geographic position of Hungary within Europe. (b) Location of the 13 study sites in the Great Hungarian Plain, Eastern Hungary. (c) A kurgan and a transect layout. (d) A permanent 0.25 × 0.25 m^2^ plot with *S. nemorosa* .

The study was located in the Great Hungarian Plain in Eastern Hungary, Europe (47°19′ 32″ N—21°6′ 58″ E, Figure 1a,b). The climate is continental with mean annual temperatures of 10°C–11°C, average precipitations of 500–600 mm, and a typical elevation of 90–120 m.a.s.l. (National Meteorological Service of Hungary 2022). In general, soils are highly fertile Chernozems, making them ideal for agriculture (Deák, Bede, et al. 2021). We worked on 13 sites (11 kurgans and two reference grasslands on flat land) where *S. nemorosa* was present, spanning across 135 km (E—W) and 79 km (N—S) (Figure 1b). Kurgans were selected to represent different habitat area (basal area ranged between 528 and 4321 m^2^) and connectivity (Hanski connectivity index ranged between 0 and 338.4). *S. nemorosa* grew in remnant grassland patches dominated by *Festuca rupicola*, and other typical grasses were *Agropyron cristatus, Stipa capillata, Bromus inermis*. Because of lack of management, on kurgans patches of weeds (*Carduus* spp., *Cirsium* spp., *Rumex* spp.), native woody species ( *Crataegus monogyna* , *Prunus spinosa* ), and invasive woody species ( *Gleditsia triacanthos* , *Robinia pseudoacacia* , *Lycium barbarum* ) were typically present. The reference grasslands were usually mown in June–July.

### 2.2 Study Species

*Salvia nemorosa* L. or Woodland sage (*Lamiaceae*) is a zoogamous, short‐lived hemicryptophyte, without active seed dispersal mechanisms and without obvious clonality, typically growing 20–50 cm tall (PADAPT 2024). It has simple leaves with opposite leaf arrangement and can grow a large number of stems, most of which are usually generative in mature individuals. The inflorescence is a pseudo‐spike composed of cymose inflorescences arranged in a verticillaster. The main inflorescence generally develops side inflorescence pairs that can further produce secondary and tertiary side inflorescences, thus extending the flowering time. The species is disturbance tolerant, thermophile, halophobe, heliophile, and xero‐tolerant, and it is characteristic of the *Salvio‐Festuceum rupicolae* loess grassland association (Borhidi 1995). It is a European species with a Pontic—Mediterranean—Central European range, where it is normally exposed to severe fluctuations in rainfall distribution and high temperatures (PADAPT 2024). Due to the severe decline and degradation of dry grasslands, *S. nemorosa* has often found refuge in small habitat islands such as kurgans and roadside verges over large parts of its distribution area (Deák et al. 2018; Moysiyenko and Sudnik‐Wójcikowska 2010; Valkó et al. 2018).

### 2.3 Data Collection

Data collection followed the protocol of Plantpopnet, a spatially distributed model system for population ecology (Buckley et al. 2019). Data was collected at peak flowering of *S. nemorosa* in June–July, over three consecutive years between 2021 and 2023. Eight sites were censused each year throughout the length of the study, and a further four sites were censused only twice as new sites were added to the study between 2022 and 2023, while one site was only censused once in 2021 due to subsequent census difficulties. We established up to four permanent transects of varying lengths (4–10 m), depending on the area of the habitat patch, where the number of individuals was representative of the habitat (Figure 1c). On kurgans, *S. nemorosa* individuals were often limited to only one side of the kurgan (typically on South‐Southwestern slopes or on the top), but on three kurgans, a larger number of individuals allowed setting up transects on two contrasting slopes. Along each transect, we laid down permanent contiguous plots of 0.5 × 0.5 m, within which we permanently marked all *S. nemorosa* individuals with a numbered linoleum tag, including new individuals each consecutive year, and if there were no individuals, the plot was marked as empty (Figure 1d). We recorded site (GPS coordinates, date of visit, management, habitat type) and transect level (cardinal aspect and slope) information. For each plant, we recorded in situ three vegetative traits: height of the tallest stem (excluding the inflorescence), number of stems, length, and width of the two biggest leaves forming a leaf pair, and two reproductive traits: inflorescence length and number of primary side inflorescence pairs. To calculate mean leaf area, we approximated the measured leaf shapes to a triangle, and we multiplied the leaf length and width, which we averaged across the two measured leaves.

### 2.4 Data Selection

In this study, we narrowed our analyses only to mature plants, to capture maximum attainable growth responses that can be related to the reproductive effort of the same individuals. We defined mature individuals as plants that had a flowering probability (i.e., the likelihood that a plant develops inflorescence) higher than 50%. For this, we modeled the flowering state (yes or no) as a function of stem height, by fitting a Generalized Linear Mixed‐Effects Model (GLMM) with binomial error distribution in the lme4 package in R (Bates et al. 2015). Fixed effects were the tallest stem height in interaction with study year, while study site, transect, plot, and plant were nested random effects. We tested the model assumptions using the DHARMa package (Hartig 2022). We then used the generic function *predict* to obtain the flowering probability values for each individual, and individuals predicted a flowering probability lower than 50% were removed from further analyses. The model structure and results are presented in the Supporting material (Figure S1, Table S5).

To ensure data quality, we removed imperfect observations e.g., dry plants or inflorescences, extreme values due to unusual growing circumstances, etc. After this step, the final number of plants involved in the models was 569 mature individuals, ranging between 13 and 49 (2021), 12–67 (2022), and 19–71 (2023) plants per site and per year.

### 2.5 Landscape Structure and Microclimate

We used the connectivity index by Hanski et al. (2000) to quantify the level of habitat isolation within a 300 m buffer around the focal habitat patch (a lower value indicating higher isolation):

$${\mathrm{CI}}_{i}=\sum_{\mathrm{ij}=1}^{n}\exp\left(-\propto d_{\mathrm{ij}}\right)A_{j}^{\beta },$$

where *A*_*j*_ is the area of a neighboring grassland within the 300 m buffer area, *d*_*ij*_ is the distance from the focal patch to the neighboring grassland, *α* is a species‐specific parameter related to the species' dispersal ability, and *β* a parameter describing the scaling of immigration, both set to 5 based on the assumption of a relatively low dispersal of 200 m for *S. nemorosa* . The area of the neighboring grassland was calculated from digitized present day (2014–2016) habitat maps (source: Unified National Projection System, 1:10,000 topographic map of Hungary [Institute and Museum of Military History, Budapest]), Satellite images (Google Maps) and field surveys, as in Deák, Rádai, et al. (2021).

We calculated the habitat area as the basal area of the kurgan or the area of the grassland patch using the same method as described above (Deák, Rádai, et al. 2021). To ease calculations due to the disproportionately large area of one of the reference grasslands compared to the area of the kurgans, we unified the Hanski connectivity and habitat area values for the two reference grasslands to the value of the grassland with the smaller area (232,061 m^2^).

To characterize the microclimate of each transect we used the heat load index, a direct measure of incident solar radiation on a land surface (Buttrick et al. 2015) that combines aspect (converted into degrees, slope, and latitude values; McCune and Keon 2002), and which we calculated with a function by Zelený and Lin (2019) in R, using the first equation:

$$\text{Heat load}=e^{\left(-1.467+1.582*\cos\left(L\right)*\cos\left(S\right)-1.500*\cos\left(A\right)*\sin\left(S\right)*\sin\left(L\right)-0.262*\sin\left(L\right)*\sin\left(S\right)+0.607*\sin\left(A\right)*\sin\left(S\right)\right)},$$

where *L* is the site latitude, *A* is the aspect where the *S. nemorosa* populations were found, and *S* is the slope angle of the transect. The heat load value calculated this way represented a coarse, transect‐level value which could not describe the fine‐scale variation of climatic conditions within microhabitats.

### 2.6 Data Analysis

We fitted Generalized Mixed‐Effects models (GLMM) using the glmmTMB package in R (Brooks et al. 2017; R Core Team 2021) to test the effect of isolation, habitat area, heat load, and study year on five measured traits of *S. nemorosa* (tallest stem height, number of stems, mean leaf area, main inflorescence length, and number of primary side inflorescence pairs). The vegetative trait models had the Hanski index, habitat area, heat load, and study year specified as fixed effects, while in the reproductive trait models, the date of visit was an additional fixed effect to cover the variance due to the continuous growth of the open inflorescence. Study site, transect, plot, and plant were nested random effects in all models to avoid pseudoreplication due to repeated measurements (but plot was removed from some models due to overly complex model structure). We fitted models of stem height and leaf area with Gaussian error distribution with identity link, models of the number of stems with negative binomial error distribution with log link, models of inflorescence length with tweedie error distribution with log link (preferred due to flexibility in handling occasional large values), while models of the number of primary side inflorescences had Conway–Maxwell Poisson error distribution with log link and the zero‐inflation term specified. Due to dealing with only two reference grasslands which had a very large area compared to the kurgans, we fitted all models on two separate datasets: (a) kurgans‐only and (b) kurgans and reference grasslands, referred to hereafter as “all sites” This way we kept the structure of the models relatively simple and avoided model singularity due to the low number of reference grasslands. In the main text, we highlight the significant relationships obtained using both datasets, and we present the model structure and results for the kurgans‐only dataset in Table 2 and for the “all sites” dataset in Table S2.

**TABLE 2:** Details of linear mixed‐effects models (LMMs) testing the effect of landscape structure, microclimate, and study year on five *Salvia nemorosa* traits using data collected on 11 kurgans.

Response variable and model structure	Explanatory variables	Estimate (*β*)	SE (*β*)	*p*	*R* ^2^	*p* (K.S. test)
Stem height *glmmTMB (log(stem_hei_noinf_allplants) ~ hload_sc + Hanski_sc + log_habitat_area_sc + c_year + (1|site_id/trans_id/plant_id), data = stemhe_noNA2, REML = TRUE, control = glmmTMBControl (optCtrl = list (iter.max = 1e3, eval.max = 1e3)), family = gaussian (link = “identity”))*	*(Intercept)*	*5.950*	*0.046*	*< 0.001*	*R* ^2^c = 0.756 *R* ^2^m = 0.543	0.082
	**Heat load**	**−0.175**	**0.030**	**< 0.001**		
	Hanski index	0.034	0.050	0.368		
	Kurgan area	−0.005	0.051	0.942		
	**Year 2022**	**−0.367**	**0.019**	**< 0.001**		
	**Year 2023**	**0.094**	**0.018**			
Mean leaf area *glmmTMB (log(mean_leafarea) ~ hload_sc + Hanski_sc + log_habitat_area_sc + c_year + (1|site_id/trans_id/plant_id), data = leafarea_noNA2, REML = TRUE, control = glmmTMBControl (optCtrl = list (iter.max = 1e3, eval.max = 1e3)), family = gaussian (link = “identity”))*	*(Intercept)*	*7.641*	*0.069*	*< 0.001*	*R* ^2^c = 0.482 *R* ^2^m = 0.207	0.088
	**Heat load**	**−0.177**	**0.057**	**0.002**		
	Hanski index	0.090	0.073	0.128		
	Kurgan area	0.015	0.075	0.675		
	**Year 2022**	**−0.255**	**0.043**	**< 0.001**		
	**Year 2023**	**0.200**	**0.041**			
Number of stems *glmmTMB (num_stems ~ hload_sc + Hanski_sc + log_habitat_area_sc + c_year + (1|site_id/trans_id/plant_id), data = numstems_noNA2, REML = TRUE, control = glmmTMBControl (optCtrl = list (iter.max = 1e3, eval.max = 1e3)), family = nbinom2 (link = “log”))*	*(Intercept)*	*2.147*	*0.050*	*< 0.001*	*R* ^2^c = 0.852 *R* ^2^m = 0.095	0.082
	**Heat load**	**−0.180**	**0.043**	***< 0.001***		
	**Hanski index**	**0.218**	**0.045**	**< 0.001**		
	Kurgan area	−0.009	0.042	0.815		
	**Year 2022**	**−0.155**	**0.044**	**< 0.001**		
	**Year 2023**	**−0.367**	**0.043**			
Inflorescence length *glmmTMB (inf_len ~ hload_sc + Hanski_sc + habitat_area_sc + date_number_sc + c_year + (1|site_id/trans_id/plot_id/plant_id), data = inflen2_large, REML = TRUE, control = glmmTMBControl (optCtrl = list (iter.max = 1e3, eval.max = 1e3)), family = tweedie (link = “log”))*	*(Intercept)*	*4.881*	*0.060*	*< 0.001*	*R* ^2^c = 0.497 *R* ^2^m = 0.245	0.145
	Heat load	−1.441	4.706	0.969		
	Hanski index	−0.031	0.044	0.064		
	Kurgan area	0.022	0.055	0.625		
	Date of visit	−0.008	0.026	0.587		
	**Year 2022**	**−0.505**	**0.052**	**< 0.001**		
	**Year 2023**	**−0.103**	**0.055**			
Number of primary side inflorescence pairs *glmmTMB (prim_side_inf_pairs ~ hload_sc + Hanski_sc + log_habitat_area_sc + date_number_sc + c_year + (1|site_id/trans_id/plot_id/plant_id), data = sideinf2_large, REML = TRUE, control = glmmTMBControl (optCtrl = list (iter.max = 1e3, eval.max = 1e3)), ziformula = ~1, family = compois (link = “log”))*	*(Intercept)*	*0.090*	*0.184*	*< 0.001*	*R* ^2^c = 0.397 *R* ^2^m = 0.213	0.847
	**Heat load**	**−0.359**	**0.122**	**0.006**		
	Hanski index	0.112	0.180	0.523		
	Kurgan area	0.136	0.169	0.293		
	**Date of visit**	**0.200**	**0.070**	**0.005**		
	**Year 2022**	**−0.865**	**0.156**	**0.001**		
	**Year 2023**	**−0.150**	**0.158**			

*Note:* The first two columns show the response variable and the model structure, and the explanatory variables, respectively. The next columns show the coefficient means (β) and standard errors SE (β), the *p* value determined by ANOVA tests, the conditional (*R* ^2^c) and marginal (*R* ^2^m) R squared values of the model, and the *p* values corresponding to the Kolmogorov–Smirnov test (K.S.) of model fit. Significant effects are shown in bold letters.

Because habitat area and connectivity were correlated (Figure S2), we refitted the models with either habitat area or Hanski index excluded from both datasets (Table S3, Table S4). This approach allowed us to identify uncertain area or connectivity effects due to the presence of both terms in the main models.

Finally, to investigate whether microclimate effects were modulated by between‐year weather variation, we refitted the models to test the effect of the interaction between heat load and study year on the measured traits. Because of increased model complexity causing nonconvergence warnings, we omitted the Hanski index, habitat area, and date of visit from this set of models, while keeping the rest of the model structure unchanged. For this analysis, we used the kurgans‐only dataset, due to uniform habitat conditions producing more robust models (Table 3).

**TABLE 3:** Details of linear mixed‐effects models (LMMs) testing the interaction between heat load (microclimate) and study year (weather proxy) on five *Salvia nemorosa* traits using data collected on 11 kurgans.

Response variable and model structure	Explanatory variables	Estimate (β)	SE (β)	*p*	*R* ^2^	*p* (K.S. test)
Stem height glmmTMB (log(stem_hei_noinf_allplants) ~ hload_sc * c_year + (1|site_id/trans_id/plot_id/plant_id), data = stemhe_noNA2, REML = TRUE, control = glmmTMBControl (optCtrl = list (iter.max = 1e3, eval.max = 1e3)), family = gaussian (link = “identity”))	*(Intercept)*	*5.924*	*0.042*	*< 0.001*	*R* ^2^c = 0.760 *R* ^2^m = 0.565	0.127
	**Heat load**	**−0.226**	**0.031**	**< 0.001**		
	**year 2022**	**−0.342**	**0.020**	**< 0.001**		
	**year 2023**	**0.114**	**0.019**			
	**Heat load: year 2022**	**0.052**	**0.019**	**< 0.001**		
	**Heat load: year 2023**	**0.074**	**0.019**			
Mean leaf area glmmTMB (log(mean_leafarea) ~ hload_sc * c_year + (1|site_id/trans_id/plot_id/plant_id), data = leafarea_noNA2, REML = TRUE, control = glmmTMBControl (optCtrl = list (iter.max = 1e3, eval.max = 1e3)), family = gaussian (link = “identity”))	*(Intercept)*	*7.646*	*0.065*	*< 0.001*	*R* ^2^c = 0.476 *R* ^2^m = 0.219	0.134
	**Heat load**	**−0.131**	**0.060**	**< 0.001**		
	**year 2022**	**−0.270**	**0.045**	**< 0.001**		
	**year 2023**	**0.178**	**0.043**			
	**Heat load: year 2022**	**−0.103**	**0.042**	**< 0.001**		
	**Heat load: year 2023**	**−0.021**	**0.043**			
Number of stems glmmTMB (num_stems ~ hload_sc * c_year + (1|site_id/trans_id/plot_id/plant_id), data = numstems_noNA2, REML = TRUE, control = glmmTMBControl (optCtrl = list (iter.max = 1e3, eval.max = 1e3)), family = nbinom2 (link = “log”))	*(Intercept)*	*2.078*	*0.074*	*< 0.001*	*R* ^2^c = 0.840 *R* ^2^m = 0.066	0.082
	**Heat load**	**−0.258**	**0.071**	**< 0.001**		
	**year 2022**	**−0.123**	**0.045**	**< 0.001**		
	**year 2023**	**−0.347**	**0.044**			
	**Heat load: year 2022**	**0.050**	**0.041**	**< 0.001**		
	**Heat load: year 2023**	**0.271**	**0.044**			
Inflorescence length glmmTMB (inf_len ~ hload_sc * c_year + (1|site_id/trans_id/plot_id/plant_id), data = inflen2_large, REML = TRUE, control = glmmTMBControl (optCtrl = list (iter.max = 1e3, eval.max = 1e3)), family = tweedie (link = “log”))	*(Intercept)*	*4.895*	*0.060*	*< 0.001*	*R* ^2^c = 0.505 *R* ^2^m = 0.243	0.087
	**Heat load**	**0.037**	**0.051**	**0.003**		
	**year 2022**	**−0.521**	**0.051**	**< 0.001**		
	**year 2023**	**−0.110**	**0.044**			
	**Heat load: year 2022**	**−0.131**	**0.050**	**0.001**		
	**Heat load: year 2023**	**0.009**	**0.048**			
Number of primary side inflorescence pairs glmmTMB (prim_side_inf_pairs ~ hload_sc * c_year + (1|site_id/trans_id/plant_id), data = sideinf2_large, REML = TRUE, control = glmmTMBControl (optCtrl = list (iter.max = 1e3, eval.max = 1e3)), ziformula = ~1, family = compois (link = “log”))	*(Intercept)*	*0.232*	*0.185*	*< 0.014*	*R* ^2^c = 0.405 *R* ^2^m = 0.268	0.435
	**Heat load**	**−0.440**	**0.147**	**< 0.004**		
	**year 2022**	**−0.977**	**0.168**	**< 0.001**		
	**year 2023**	**−0.375**	**0.138**			
	**Heat load: year 2022**	**−0.202**	**0.136**	**< 0.001**		
	**Heat load: year 2023**	**0.410**	**0.134**			

*Note:* The first two columns show the response variable and the model structure, and the explanatory variables respectively. The next columns show the coefficient means (β) and standard errors SE (β), the *p* value determined by ANOVA tests, the conditional (*R* ^2^c) and marginal (*R* ^2^m) R squared values of the model and the *p* values corresponding to the Kolmogorov–Smirnov test (K.S.) of model fit. Significant effects are shown in bold letters.

We tested if residuals followed the expected distribution using the Kolmogorov–Smirnov test, and the diagnostics of overdispersion and zero inflation using the DHARMa package in R (Hartig 2022). We explored the model goodness‐of‐fit by calculating Nakagawa's conditional and marginal R^2^ values, using the *performance::r2* command in the *performance* package in R (Lüdecke et al. 2021). Because we had a priori expectations about the effect of explanatory variables, we employed a hypothesis testing approach. We examined whether the effect of explanatory variables was significant by comparing the full models encompassing all variables with reduced models from which the variable of interest was removed, using ANOVA (Likelihood Ratio) tests. To make the effect size of variables comparable within each set of models, all continuous predictor variables were centered on 0 and scaled to have unit variance. In models fitted with Gaussian error distribution, the response variables were log‐transformed to improve the normality of residuals.

## 3 Results

The descriptive statistics for the analyzed dataset are presented in Table S1.

**FIGURE 2:**
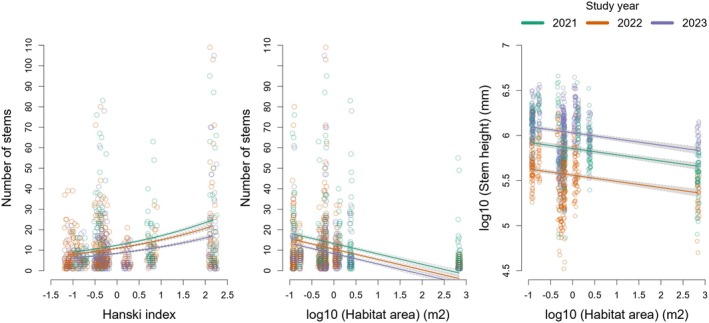
Scatterplots with fitted lines showing significant effects of landscape structure on traits of *Salvia nemorosa* , as detected by linear mixed‐effects models: The effect of isolation on the number of stems in a dataset of 11 kurgans (first panel), and the effect of habitat area on the number of stems (second panel), and the effect of habitat area on the stem height without inflorescence (third panel) respectively in a dataset including 11 kurgans and two reference flat grasslands. The fitted lines are shown separately for each study year, but models did not test for interactions. X axis shows values scaled to mean and unit variance.

The Hanski connectivity index was moderately positively and significantly correlated with the number of stems in both datasets (Figure 2, Table 2). In models refitted without habitat area in the kurgans‐only dataset, the Hanski connectivity index was still positively and significantly correlated with the number of stems, indicating an unequivocal effect of isolation (kurgans‐only dataset, Table S3).

Habitat area had a moderately negative, significant effect on the number of stems and stem height, but the effects were detectable only in the dataset including all sites and were due to a comparatively lower number of stems and shorter stems in the two large reference grasslands (Figure 2, Table S2). However, in models refitted without the Hanski index, the correlation between habitat area and the number of stems was not maintained, indicating that isolation contributed to some extent to this relationship. Instead, the negative significant effect of habitat area on the stem height was maintained, indicating the effect of area independent of isolation (all sites dataset, Figure 2, Table S4).

There was a strong negative, significant effect of heat load on all measured traits except for the inflorescence length in models using both datasets (Table 2, Table S2).

Study year had a significant effect on all measured traits in models using both the kurgans‐only and the “all sites” datasets. Compared to the study year 2021, the dry 2022 year had a strong negative, significant effect on all traits, with a subsequent recovery during 2023 except for the number of stems, which lowered even further (Table 2, Table S2).

**FIGURE 3:**
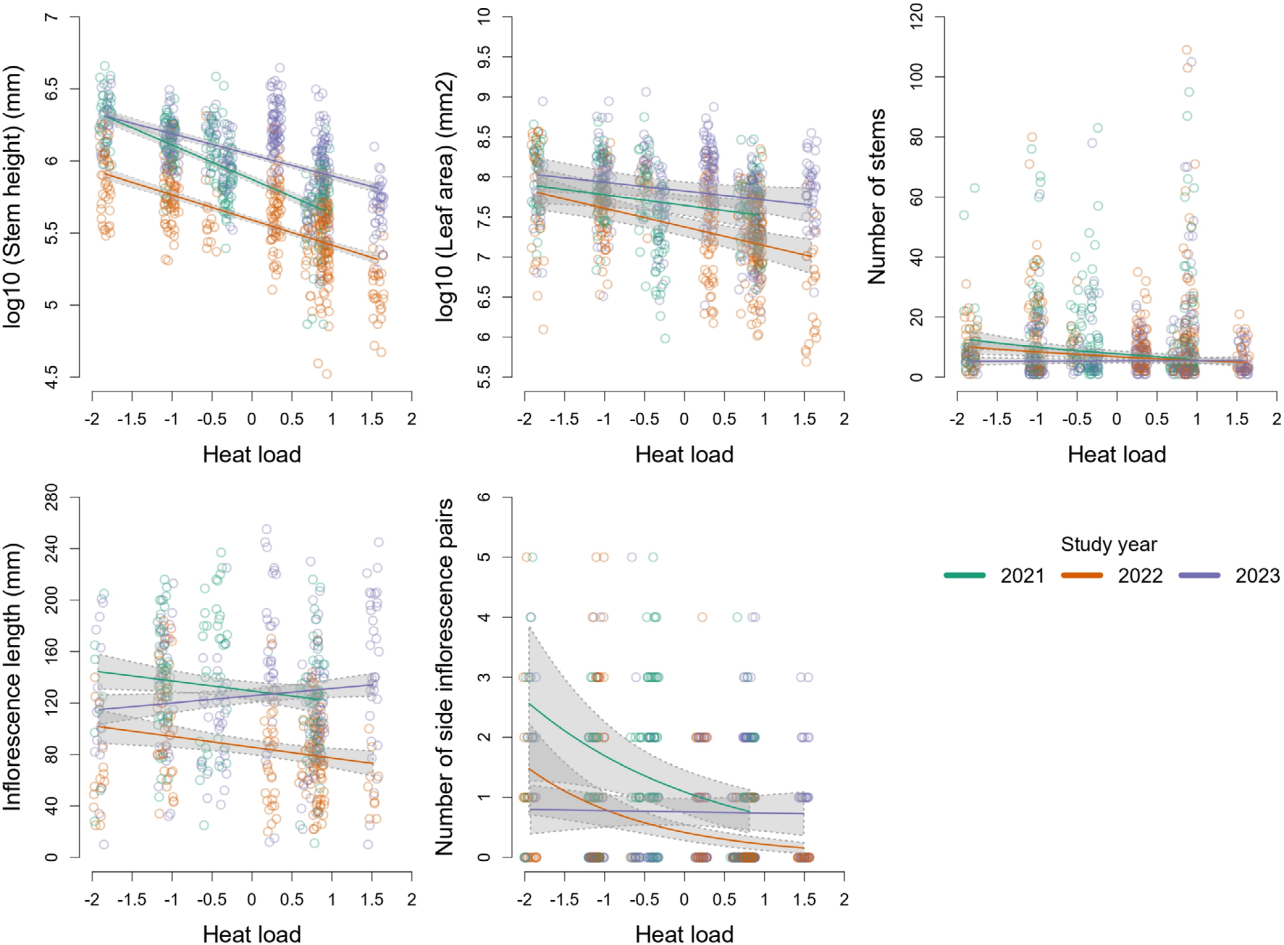
Scatterplots with fitted lines showing the significant interactions between microclimate (heat load) and study year as a proxy for regional weather conditions, as detected by linear mixed‐effects models testing the sources of variation in three vegetative and two reproductive traits of *Salvia nemorosa* measured on 11 kurgans. X axis shows values scaled to mean and unit variance.

In models testing the interaction between heat load and study year, the main effect of heat load was negative and significant for all traits except the inflorescence length, where the effect was positive, while the main effect of the year was similar to the effects detected in the other models. The interaction between the local heat load and the study year was significant in models of each trait: in the dry year 2022, the negative effect of the heat load weakened for the stem height and number of stems, which were suppressed along the entire gradient, and it strengthened for the leaf area and number of side inflorescences; in the subsequent year 2023, the negative effect of the heat load was further weakened, in particular, for the number of stems and number of side inflorescences, which were suppressed along the entire heat load gradient, and the positive effect of the heat load was strengthened for inflorescence length (Figure 3, Table 3).

**FIGURE 4:**
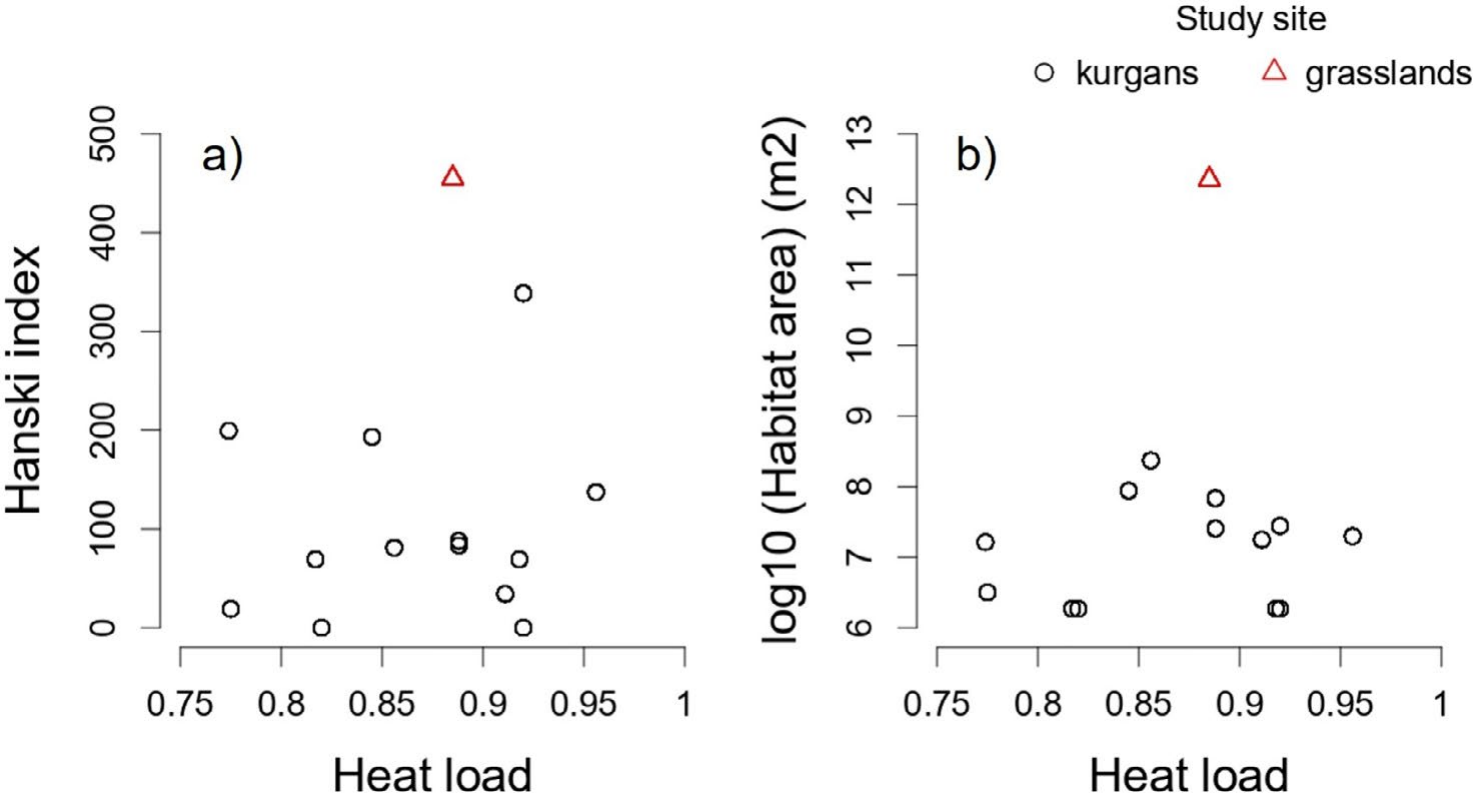
Scatterplots showing the relationship between microclimate (heat load) and Hanski index (a) and heat load and habitat area (b) on transects set up on 11 kurgans and two reference flat grasslands. Populations subject to different degrees of isolation and habitat area were positioned randomly along the heat load gradient. X axis shows values scaled to mean and unit variance.

There was no relationship between heat load and Hanski index and heat load and habitat area, thus habitats with different degrees of isolation and area were positioned randomly along the heat load gradient (Figure 4).

## 4 Discussion

Using a spatially and temporally replicated dataset in combination with a local heat load gradient we showed that the strong heat load gradient modulated by temporal fluctuations in weather conditions was a dominant driver of trait variation in *S. nemorosa* , and for some traits, these effects were superimposed on constraints due to land use changes. We propose that effects of microhabitat exposure to excessive heat load amplified by dry years may complicate the detection of the real effect of altered landscape structure on intraspecific trait variation.

### 4.1 Effect of Habitat Area and Isolation

We found intraspecific trait variation associated with constraints from isolation and small area of habitat patches in our system. On isolated kurgans, *S. nemorosa* individuals had a lower number of stems compared to kurgans benefiting from higher connectivity, although the models explained a low proportion of variation in this trait. The poorer performance of *S. nemorosa* in isolation was in line with lower seed germination rates of the species in the same habitats, indicative of genetic deterioration due to severed connectivity (Szász 2022). This is an important limitation with potential negative consequences for the future dynamics of isolated *S. nemorosa* populations because the number of stems determines major vital rates in plants in general, and in this species (Zayani 2024), and constraints on plant size could be early warning signals of population declines typical of remnant habitat fragments (Deák, Rádai, et al. 2021). Individuals had significantly taller stems on kurgans compared to large reference grasslands, suggesting that processes associated with small area affect the studied species on all kurgans. The result does not clearly support the expectation of impeded growth due to genetic deterioration in small habitat fragments (Lowe et al. 2005; Zambrano et al. 2019); instead, it aligns with responses to increased levels of competition in small habitat patches, which may cause a shift towards more acquisitive strategies in plants (Botta‐Dukát 2021). Moderate competitors such as *S. nemorosa* (Kelemen et al. 2013) may be expected to respond readily to increased levels of competition by growing taller stems, and community‐level studies confirm shifts towards taller species in the system (Deák et al. 2024). We further expected that hemicryptophytes such as *S. nemorosa* could respond to increased competition by increased investment in self‐maintenance favoring local persistence by, e.g., increased bud bank on the rhizome or shift to large sizes (Biddick et al. 2019; Zambrano et al. 2019); however, in *S. nemorosa*, this is a less likely cause of the increased number of stems on kurgans. Because both area effects were solely driven by the two reference grasslands having individuals with significantly fewer and shorter stems, the management history of the sites is more likely the trigger of the responses. Lack of disturbance, typical of abandoned grasslands, enables forbs to grow a larger number of stems and reach higher local abundance, while frequent mowing, a regular management practice in extensive grasslands, may reset the life cycle of individuals at critical life stages, reducing the biomass (Csergő et al. 2013; Dee and Baum 2019). As most kurgans are unmanaged, this may on the one hand increase levels of competition, but, on the other hand, leave *S. nemorosa* individuals time to reach sizes closer to their biological schedules.

### 4.2 Effects of Microhabitat Heat Load

We found strong negative responses in all traits to increased heat load. This result aligns with abundant evidence of heat stress having negative impacts on plant growth and reproduction (Descamps et al. 2021; Tene et al. 2023; Willi and van Buskirk 2022), and mirrors the interspecific patterns of trait distributions in the same system (Deák et al. 2024; Deák, Bede, et al. 2021). The trait distribution in *S. nemorosa* followed expectations from the “species interactions—abiotic stress” hypothesis, which provides a theoretical framework to explain limits to species distribution along a low stress (high competition)—high stress (low competition) gradient (Kirk et al. 2021; Louthan et al. 2015). *S. nemorosa* individuals occurred between relatively lower heat load conditions (northerly, “cooler” side of kurgans) and high heat load conditions (southerly, “hotter” side of kurgans). This gradient causes whole community level shifts from strong competitor grasses and taller forbs to stress‐tolerant plants or plants with shorter life cycles on kurgans (Deák et al. 2024). Along this gradient, as a balanced CSR strategist species (Kelemen et al. 2013), *S. nemorosa* shifted from competitive avoidance and fast resource acquisition responses under low heat load conditions (tall stems, large leaf area, several side inflorescences) (Botta‐Dukát 2021), towards stress tolerance and self‐maintenance responses under high heat load conditions (shorter stems, small leaf area, few side inflorescences). Each type of response may have cascading effects on the persistence strategies of the species, for example, a higher number of side inflorescences under more competitive conditions could enable a bet‐hedging strategy extending the chances of pollination and seed maturation and dispersal, while a lower number of side inflorescences and a shorter inflorescence under more stressful conditions could enable a faster life cycle with fast seed development and maturation. Our result thus underscores the importance of local processes favoring or jeopardizing the persistence of individuals in remnant population fragments (Dupré and Ehrlén 2002; Griffen and Drake 2008; Ibáñez et al. 2014; Marini et al. 2012; Xu et al. 2017). We further advance that trait shifts expected as a result of the modified landscape structure may have been blurred by the strong responses to the heat stress gradient typical of kurgans (Deák et al. 2024), because populations subject to different degrees of isolation and habitat area were positioned randomly along the heat load gradient.

### 4.3 Effects of Between‐Year Weather Variation

Our approach provided a unique opportunity to study how trait responses to the strong heat load gradient may be modulated by annual weather conditions, and we found that the effects of the heat load on traits were strongly dependent on the study year. Across the study interval, the year 2022 was the hottest and driest in the studied region. For example, in June, the month of peak flowering of *S. nemorosa* , average temperatures exceeded the 1991–2020 average temperatures by 2°C–3°C, and total June precipitation was only 30%–40% of normal decadal values. By comparison, the June of the year 2021 was 1°C–2°C warmer and 20%–30% drier than the previous decade, while the June of the year 2023 was 0°C–0.5°C cooler and 100%–120% wetter than the previous decade (Hungarian Meteorological Service, https://www.met.hu/). Weather conditions are known to synchronize fluctuations of spatially separated populations (Moran 1953; Salguero‐Gómez and Gamelon 2021), which was the case in the extremely dry year 2022 that exacerbated the negative heat load effects in all traits. In small populations (or subpopulations) occupying the stressful end of the gradient, such years may have catastrophic consequences (Grear and Burns 2007; Salguero‐Gómez and Gamelon 2021). In light of the predicted increasing frequency of dry years, the long‐term fate of these sub(populations) may become uncertain (Orbán et al. 2023; Spinoni et al. 2018). However, more favorable years (lower temperatures and higher precipitation periods) desynchronized the response of different traits to the heat stress gradient. While some traits remained unresponsive to the heat load gradient, the relationship between heat load and inflorescence length response reversed, switching from negative to positive. Interactive effects of habitat quality and climate on plant traits are likely frequent yet largely underexplored (Nicolè et al. 2011), and the amount of noise induced in the system challenges approaches of snapshot trait surveys for describing land use change effects on plant populations. Consequently, long‐term observations across environmental stress gradients are necessary to capture the relative contribution of natural and human stressors to the spatial patterns of plant traits in structured landscapes (Buckley and Puy 2022; Compagnoni et al. 2024).

## 5 Conclusions

Our results suggest that intraspecific trait variation along local heat stress gradients may interfere with plant responses to landscape reconfiguration, challenging our ability to detect important human footprint effects on plant populations. In particular, the role of dry years in altering the effects of local exposure to heat is critical in populations confined to remnant habitat fragments. Robust predictive ecological models of intraspecific trait variation in human‐altered landscapes should consider not only landscape structure effects but also the availability of heterogeneous microclimates capable of buffering negative effects of weather extremes.

## Author Contributions

**Santiago Ordonez:** data curation (lead), formal analysis (lead), project administration (supporting), resources (equal), visualization (lead), writing – original draft (lead). **Balázs Deák:** conceptualization (supporting), formal analysis (supporting), investigation (supporting), methodology (supporting), resources (supporting), validation (supporting), writing – review and editing (equal). **Orsolya Valkó:** conceptualization (supporting), investigation (supporting), methodology (supporting), resources (supporting), validation (supporting), writing – review and editing (equal). **Krisztina Verbényiné Neumann:** data curation (supporting), formal analysis (supporting), investigation (supporting), resources (supporting), writing – review and editing (supporting). **Vivien Szász:** data curation (supporting), investigation (supporting), resources (equal), validation (supporting), writing – review and editing (supporting). **Anna Mária Csergő:** conceptualization (lead), data curation (equal), formal analysis (equal), funding acquisition (lead), investigation (lead), methodology (lead), project administration (lead), resources (equal), supervision (lead), validation (equal), visualization (supporting), writing – original draft (equal), writing – review and editing (lead).

## Conflicts of Interest

The authors declare no conflicts of interest.

## Supporting information


**Figure S1.** Plot showing the modeled *Salvia nemorosa* flowering probability (model details are presented in Table S5).
**Figure S2.** Scatterplot showing the correlation between the area and Hanski connectivity index of the studied habitat fragments.
**Table S1.** Descriptive statistics of site conditions and *Salvia nemorosa* traits at the studied sites.
**Table S2.** Details of models testing the effect of landscape structure, heat load (microclimate) and study year (weather proxy) on five *Salvia nemorosa* traits using data collected on 11 kurgans and two flat reference grasslands (all sites). The first three columns show the response variable, the model structure, and the explanatory variables respectively. The next columns show the coefficient means (β) and standard errors SE (β), the *p* value determined by ANOVA tests, the conditional (*R* ^2^c) and marginal (*R* ^2^m) R squared values of the model and the *p* values corresponding to the Kolmogorov–Smirnov test (KS) of model residuals distribution. Significant effects are shown in bold letters.
**Table S3.** Details of models of *Salvia nemorosa* traits from which either area or Hanski index were removed, using data collected on 11 kurgans (kurgans‐only dataset). The first column shows the response variable, the second and third columns indicate the model structure, the fourth column indicates the explanatory variables. The next columns show the coefficient means (β) and standard errors SE (β), the *p* value determined by ANOVA tests, the conditional (*R* ^2^c) and marginal (*R* ^2^m) R squared values of the model and the *p* values corresponding to the Kolmogorov–Smirnov test (KS) of model residuals distribution. Significant effects are shown in bold letters.
**Table S4.** Details of models of *Salvia nemorosa* traits from which either area or Hanski index were removed, using data collected on 11 kurgans and two flat reference grasslands (“all sites” dataset). The first column shows the response variable, the second and third columns indicate the model structure, the fourth column indicates the explanatory variables. The next columns show the coefficient means (β) and standard errors SE (β), the *p* value determined by ANOVA tests, the conditional (*R* ^2^c) and marginal (*R* ^2^m) R squared values of the model and the *p* values corresponding to the Kolmogorov–Smirnov test (KS) of model residuals distribution. Significant effects are shown in bold letters.
**Table S5.** Details of the model of *Salvia nemorosa* flowering probability. The first three columns show the response variable, the model structure, and the explanatory variables respectively. The next columns show the coefficient means (β) and standard errors SE (β), the *p* value determined by ANOVA tests, the conditional (*R* ^2^c) and marginal (*R* ^2^m) R squared values of the model and the *p* values corresponding to the Kolmogorov–Smirnov test (KS) of model residuals distribution. Significant effects are shown in bold letters.

## Data Availability

Data and the R script are available in the Dryad data repository: https://doi.org/10.5061/dryad.hmgqnk9th.
