# Supplementary material for: Microclimate and Dry Years Interfere With Landscape Structure Effects on Intraspecific Trait Variation
Source: Ecol Evol. 2025 May 13;15(5):e71417. doi: 10.1002/ece3.71417 (PMC12074717; doi:10.1002/ece3.71417)
Supplement: Supplementary file 1 — Figure S1. Plot showing the modeled Salvia nemorosa flowering probability (model details are presented in Table S5). Figure S2. Scatterplot showing the correlation between the area and Hanski connectivity index of the studied habitat fragments. Table S1. Descriptive statistics of site conditions and Salvia nemorosa traits at the studied sites. Table S2. Details of models testing the effect of landscape structure, heat load (microclimate) and study year (weather proxy) on five Salvia nemorosa traits using data collected on 11 kurgans and two flat reference grasslands (all sites). The first three columns show the response variable, the model structure, and the explanatory variables respectively. The next columns show the coefficient means (β) and standard errors SE (β), the p value determined by ANOVA tests, the conditional (R 2c) and marginal (R 2m) R squared values of the model and the p values corresponding to the Kolmogorov–Smirnov test (KS) of model residuals distribution. Significant effects are shown in bold letters. Table S3. Details of models of Salvia nemorosa traits from which either area or Hanski index were removed, using data collected on 11 kurgans (kurgans‐only dataset). The first column shows the response variable, the second and third columns indicate the model structure, the fourth column indicates the explanatory variables. The next columns show the coefficient means (β) and standard errors SE (β), the p value determined by ANOVA tests, the conditional (R 2c) and marginal (R 2m) R squared values of the model and the p values corresponding to the Kolmogorov–Smirnov test (KS) of model residuals distribution. Significant effects are shown in bold letters. Table S4. Details of models of Salvia nemorosa traits from which either area or Hanski index were removed, using data collected on 11 kurgans and two flat reference grasslands (“all sites” dataset). The first column shows the response variable, the second and third columns indicate the model structur [file ECE3-15-e71417-s001.docx]

**SUPPORTING INFORMATION**


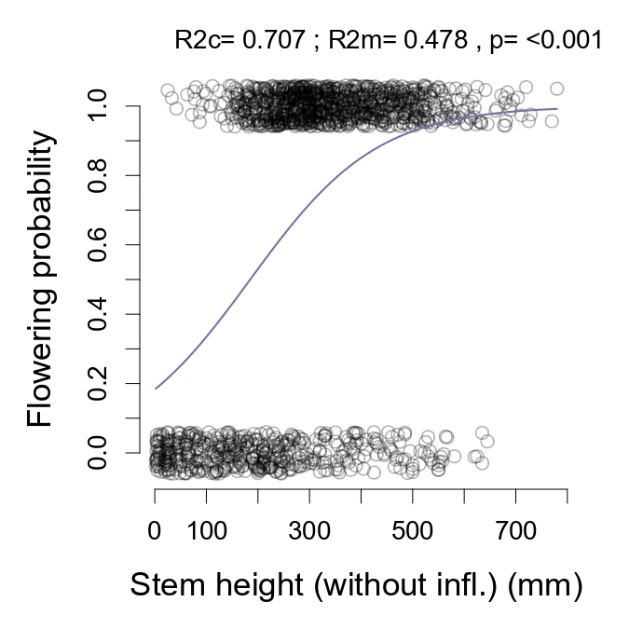


**Figure S1.** Plot showing the modelled *Salvia nemorosa* flowering probability (model details are presented in Table S5).


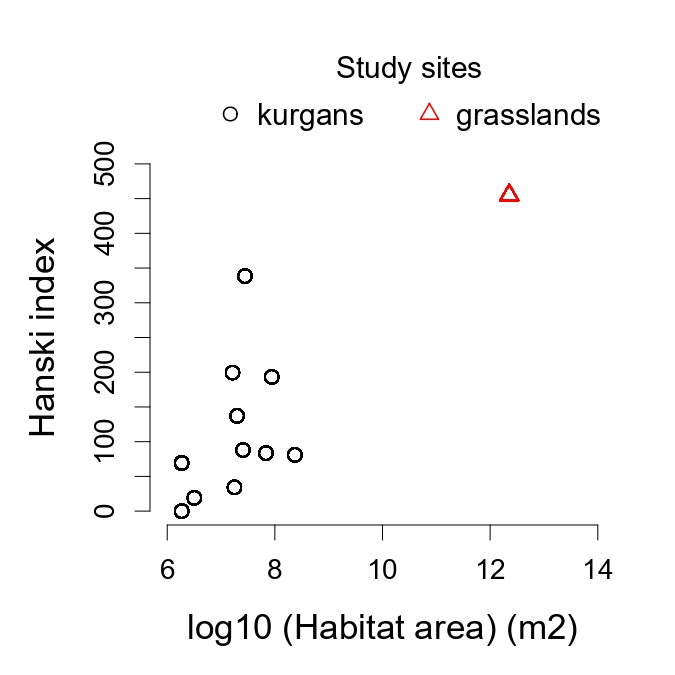


**Figure S2.** Scatterplot showing the correlation between the area and Hanski connectivity index of the studied habitat fragments.

**Table S1.** Descriptive statistics of site conditions and *Salvia nemorosa* traits at the studied sites.

| **Variable (unit)** | **Mean ± 1SD** | **Minimum** | **Maximum** |
| --- | --- | --- | --- |
| Hanski index  (without unit) | 113.02 ± 98.754 (kurgans) | 0 | 338.4 (kurgans) |
| Area (m^2^) | 1,725.727 ± 1,133.711 (kurgans) | 528 m^2^ (kurgans) | 4,321 m^2^ (kurgans) |
| Heat load (without unit) | 0.872±0.049 | 0.774 (10-degree slope, E-NE aspect) | 0.956 (15-degree slope, W-SW aspect) |
| Tallest stem height  (excluding inflorescence) (mm) | 357.525 ± 125.708 | 92 mm | 780 mm |
| Mean leaf area  (biggest leaf pair) (mm^2^) | 2,316.246 ± 1,171.383 | 250 mm^2^ | 8637 mm^2^ |
| Number of stems (count) | 10.383 ± 13.418 | 1 | 109 |
| Mean leaf length  (biggest leaf pair) (mm) | 63.847 ± 14.277 | 25 | 177.5 |
| Mean leaf width  (biggest leaf pair) (mm) | 18.591 ± 5.45 | 7.5 | 70 |
| Main inflorescence length (mm) | 107.266 ± 56.537 | 2 | 255 |
| Number of primary side inflorescence pairs (count) | 0.84 ± 1.075 | 0 | 5 |

**Table S2.** Details of models testing the effect of landscape structure, heat load (microclimate) and study year (weather proxy) on five *Salvia nemorosa* traits using data collected on 11 kurgans and two flat reference grasslands (all sites). The first three columns show the response variable, the model structure, and the explanatory variables respectively. The next columns show the coefficient means (β) and standard errors SE (β), the p value determined by ANOVA tests, the conditional (*R^2^*c) and marginal (*R^2^*m) R squared values of the model and the p values corresponding to the Kolmogorov-Smirnov test (KS) of model residuals distribution. Significant effects are shown in bold letters.

| **Response variable** | **Model structure** | **Explanatory variables** | **Estimate (β)** | **SE (β)** | **p** | ***R^2^*** | **p (K.S.)** |
| --- | --- | --- | --- | --- | --- | --- | --- |
| Stem height | *glmmTMB(log(stem_hei_noinf_allplants) ~ hload_sc + Hanski_sc + log_habitat_area_sc + c_year + (1 \| site_id/trans_id/plot_id/plant_id), data= stemhe_noNA1, REML= TRUE, control= glmmTMBControl(optCtrl= list(iter.max= 1e3,eval.max= 1e3)), family= gaussian(link= "identity"))* | *(Intercept)* | *5.903* | *0.044* | *<0.001* | *R^2^*c= 0.774; *R^2^*m= 0.562 | 0.062 |
|  |  | **Heat load** | **-0.160** | **0.027** | **<0.001** |  |  |
|  |  | Hanski index | 0.035 | 0.068 | 0.547 |  |  |
|  |  | **Habitat area** | **-0.129** | **0.063** | **0.031** |  |  |
|  |  | **year 2022** | **-0.353** | **0.018** | **<0.001** |  |  |
|  |  | **year 2023** | **0.118** | **0.017** |  |  |  |
| Mean leaf area | *glmmTMB(log(mean_leafarea) ~ hload_sc + Hanski_sc + log_habitat_area_sc + c_year + (1 \| site_id/trans_id/plot_id/plant_id), data= leafarea_noNA1, REML= TRUE, control= glmmTMBControl(optCtrl= list(iter.max= 1e3,eval.max= 1e3)), family= gaussian(link= "identity"))* | *(Intercept)* | *7.635* | *0.062* | *<0.001* | *R^2^*c= 0.466; *R^2^*m= 0.186 | 0.075 |
|  |  | **Heat load** | **-1.67** | **0.051** | **0.002** |  |  |
|  |  | Hanski index | 0.105 | 0.093 | 0.195 |  |  |
|  |  | Habitat area | -0.075 | 0.088 | 0.322 |  |  |
|  |  | **year 2022** | **-0.239** | **0.040** | **<0.001** |  |  |
|  |  | **year 2023** | **0.179** | **0.039** |  |  |  |
| Number of stems | *glmmTMB(num_stems ~ hload_sc + Hanski_sc + log_habitat_area_sc + c_year + (1 \| site_id/trans_id/plant_id), data= numstems_noNA1, REML= TRUE, control= glmmTMBControl(optCtrl= list(iter.max= 1e3,eval.max= 1e3)), family= nbinom2(link = "log"))* | *(Intercept)* | *2.099* | *0.078* | *<0.001* | *R^2^*c= 0.792; *R^2^*m= 0.094 | 0.060 |
|  |  | **Heat load** | **-0.158** | **0.059** | **0.007** |  |  |
|  |  | **Hanski index** | **0.249** | **0.113** | **0.014** |  |  |
|  |  | **Habitat area** | **-0.292** | **0.108** | **0.004** |  |  |
|  |  | **year 2022** | **-0.152** | **0.043** | **<0.001** |  |  |
|  |  | **year 2023** | **-0.369** | **0.042** |  |  |  |
| Inflorescence length | *glmmTMB(inf_len ~ hload_sc + Hanski_sc + log_habitat_area_sc + date_number_sc + c_year + (1 \| site_id/trans_id/plant_id), data= inflen1_large, REML= TRUE, control=glmmTMBControl(optCtrl=list(iter.max=1e3,eval.max=1e3)), family= tweedie(link = "log"))* | *(Intercept)* | *4.848* | *0.057* | *<0.001* | *R^2^*c= 0.399; *R^2^*m= 0.237 | 0.055 |
|  |  | Heat load | -0.013 | 0.036 | 0.817 |  |  |
|  |  | Hanski index | -0.029 | 0.081 | 0.601 |  |  |
|  |  | Habitat area | 0.070 | 0.076 | 0.270 |  |  |
|  |  | Date of visit | -0.004 | 0.024 | 0.999 |  |  |
|  |  | **year 2022** | **-0.475** | **0.049** | **<0.001** |  |  |
|  |  | **year 2023** | **-0.011** | **0.053** |  |  |  |
| Number of primary side inflorescence pairs | glmmTMB(prim_side_inf_pairs ~ hload_sc + Hanski_sc + log_habitat_area_sc + date_number_sc + c_year + (1 \| site_id/trans_id/plot_id/plant_id) , data= sideinf1_large, REML= TRUE, control= glmmTMBControl(optCtrl= list(iter.max= 1e3,eval.max= 1e3)), ziformula=~1, family= compois(link = "log")) | *(Intercept)* | *-2.767* | *0.985* | *0.005* | *R^2^*c= 0.434; *R^2^*m= 0.140 | 0.714 |
|  |  | **Heat load** | **-0.321** | **0.110** | **0.003** |  |  |
|  |  | Hanski index | 0.117 | 0.231 | 0.631 |  |  |
|  |  | Habitat area | -0.016 | 0.216 | 0.976 |  |  |
|  |  | **Date of visit** | **0.185** | **0.138** | **0.004** |  |  |
|  |  | **year 2022** | **-0.807** | **0.138** | **<0.001** |  |  |
|  |  | **year 2023** | **-0.105** | **0.145** |  |  |  |

**Table S3.** Details of models of *Salvia nemorosa* traits from which either area or Hanski index were removed, using data collected on 11 kurgans (kurgans-only dataset). The first column shows the response variable, the second and third columns indicate the model structure, the fourth column indicates the explanatory variables. The next columns show the coefficient means (β) and standard errors SE (β), the p value determined by ANOVA tests, the conditional (*R^2^*c) and marginal (*R^2^*m) R squared values of the model and the p values corresponding to the Kolmogorov-Smirnov test (KS) of model residuals distribution. Significant effects are shown in bold letters.

| **Response variable** | **Model** | | **Model structure** | **Explanatory variables** | **Estimate (β)** | **SE (β)** | **p** | ***R^2^*** | **p**  **(K.S.)** |
| --- | --- | --- | --- | --- | --- | --- | --- | --- | --- |
| Stem height | Kurgan area effect in the absence of isolation | *glmmTMB(log(stem_hei_noinf_allplants) ~ hload_sc + log_habitat_area_sc + c_year + (1 \| site_id/trans_id/plant_id), data= stemhe_noNA2, REML= TRUE, control= glmmTMBControl(optCtrl= list(iter.max= 1e3,eval.max= 1e3)), family= gaussian(link= "identity"))* | | *(Intercept)* | *5.948* | *0.046* | *<0.001* | *R^2^*c= 0.757; *R^2^*m= 0.545 | 0.210 |
|  |  |  |  | **Heat load** | **-0.172** | **0.030** | **<0.001** |  |  |
|  |  |  |  | Kurgan area | 0.004 | 0.049 | 0.905 |  |  |
|  |  |  |  | **year 2022** | **-0.366** | **0.019** | **<0.001** |  |  |
|  |  |  |  | **year 2023** | **0.094** | **0.018** |  |  |  |
|  | Isolation effect in the absence of kurgan area | *glmmTMB(log(stem_hei_noinf_allplants) ~ hload_sc + Hanski_sc + c_year + (1 \| site_id/trans_id/plant_id), data= stemhe_noNA2, REML= TRUE, control= glmmTMBControl(optCtrl= list(iter.max= 1e3,eval.max= 1e3)), family= gaussian(link= "identity"))* | | *(Intercept)* | *5.950* | *0.043* | *<0.001* | *R^2^*c= 0.753; *R^2^*m= 0.549 | 0.120 |
|  |  |  |  | **Heat load** | **-0.174** | **0.029** | **<0.001** |  |  |
|  |  |  |  | Hanski index | 0.034 | 0.046 | 0.403 |  |  |
|  |  |  |  | **year 2022** | **-0.365** | **0.019** | **<0.001** |  |  |
|  |  |  |  | **year 2023** | **0.095** | **0.018** |  |  |  |
| Mean leaf area | Kurgan area effect in the absence of isolation | *glmmTMB(log(mean_leafarea) ~ hload_sc + Hanski_sc + log_habitat_area_sc + c_year + (1 \| site_id/trans_id/plant_id), data= leafarea_noNA2, REML= TRUE, control= glmmTMBControl(optCtrl= list(iter.max= 1e3,eval.max= 1e3)), family= gaussian(link= "identity"))* | | *(Intercept)* | *7.627* | *0.072* | *<0.001* | *R^2^*c= 0.486; *R^2^*m= 0.207 | 0.001 |
|  |  |  |  | **Heat load** | **-0.177** | **0.057** | **0.002** |  |  |
|  |  |  |  | Kurgan area | 0.029 | 0.076 | 0.591 |  |  |
|  |  |  |  | **year 2022** | **-0.261** | **0.043** | **<0.001** |  |  |
|  |  |  |  | **year 2023** | **0.198** | **0.041** |  |  |  |
|  | Isolation effect in the absence of kurgan area | *glmmTMB(log(mean_leafarea) ~ hload_sc + Hanski_sc + c_year + (1 \| site_id/trans_id/plant_id), data= leafarea_noNA2, REML= TRUE, control= glmmTMBControl(optCtrl= list(iter.max= 1e3,eval.max= 1e3)), family= gaussian(link= "identity"))* | | *(Intercept)* | *7.643* | *0.067* | *<0.001* | *R^2^*c= 0.473; *R^2^*m= 0.209 | 0.090 |
|  |  |  |  | **Heat load** | **-0.173** | **0.055** | **0.002** |  |  |
|  |  |  |  | Hanski index | 0.094 | 0.069 | 0.124 |  |  |
|  |  |  |  | **year 2022** | **-0.258** | **0.043** | **<0.001** |  |  |
|  |  |  |  | **year 2023** | **0.200** | **0.041** |  |  |  |
| Number of stems | Kurgan area effect in the absence of isolation | *glmmTMB(num_stems ~ hload_sc + log_habitat_area_sc + c_year + (1 \| site_id/trans_id/plant_id), data= numstems_noNA2, REML= FALSE, control= glmmTMBControl(optCtrl= list(iter.max= 1e3,eval.max= 1e3)), family= nbinom2(link = "log"))* | | *(Intercept)* | *2.141* | *0.075* | *<0.001* | *R^2^*c= 0.853; *R^2^*m= 0.060 | 0.028 |
|  |  |  |  | **Heat load** | **-0.159** | **0.063** | **0.008** |  |  |
|  |  |  |  | Kurgan area | 0.048 | 0.079 | 0.568 |  |  |
|  |  |  |  | **year 2022** | **-0.195** | **0.029** | **<0.001** |  |  |
|  |  |  |  | **year 2023** | **-0.401** | **0.029** |  |  |  |
|  | Isolation effect in the absence of kurgan area | *glmmTMB(num_stems ~ hload_sc + Hanski_sc + c_year + (1 \| site_id/trans_id/plant_id), data= numstems_noNA2, REML= TRUE, control= glmmTMBControl(optCtrl= list(iter.max= 1e3,eval.max= 1e3)), family= nbinom2(link = "log"))* | | *(Intercept)* | *2.170* | *0.044* | *<0.001* | *R^2^*c= 0.851; *R^2^*m= 0.096 | 0.263 |
|  |  |  |  | **Heat load** | **-0.179** | **0.042** | **<0.001** |  |  |
|  |  |  |  | **Hanski index** | **0.204** | **0.045** | **<0.001** |  |  |
|  |  |  |  | **year 2022** | **-0.191** | **0.029** | **<0.001** |  |  |
|  |  |  |  | **year 2023** | **-0.397** | **0.029** |  |  |  |
| Inflorescence length | Kurgan area effect in the absence of isolation | *glmmTMB(inf_len ~ hload_sc + habitat_area_sc + date_number_sc + c_year + (1 \| site_id/trans_id/plant_id) , data= inflen2_large, REML= TRUE, control= glmmTMBControl(optCtrl= list(iter.max= 1e3,eval.max= 1e3)), family= tweedie(link = "log"))* | | *(Intercept)* | *4.881* | *0.064* | *<0.001* | *R^2^*c= 0.462; *R^2^*m= 0.219 | 0.372 |
|  |  |  |  | Heat load | -0.013 | 0.041 | 0.085 |  |  |
|  |  |  |  | Kurgan area | 0.018 | 0.052 | 0.079 |  |  |
|  |  |  |  | Date of visit | -0.008 | 0.026 | 0.579 |  |  |
|  |  |  |  | **year 2022** | **-0.502** | **0.052** | **<0.001** |  |  |
|  |  |  |  | **year 2023** | **-0.099** | **0.054** |  |  |  |
|  | Isolation effect in the absence of kurgan area | *glmmTMB(inf_len ~ hload_sc + Hanski_sc + date_number_sc + c_year + (1 \| site_id/trans_id/plant_id), data= inflen2_large, REML= TRUE, control= glmmTMBControl(optCtrl= list(iter.max= 1e3,eval.max= 1e3)), family= tweedie(link = "log"))* | | *(Intercept)* | 4.887 | 0.062 | *<0.001* | *R^2^*c= 0.484; *R^2^*m= 0.211 | 0.147 |
|  |  |  |  | Heat load | -0.005 | 0.043 | 0.988 |  |  |
|  |  |  |  | Hanski index | -0.032 | 0.060 | 0.037 |  |  |
|  |  |  |  | Date of visit | -0.009 | 0.025 | 0.390 |  |  |
|  |  |  |  | **year 2022** | **-0.507** | **0.050** | **<0.001** |  |  |
|  |  |  |  | **year 2023** | **-0.106** | **0.025** |  |  |  |
| Number of primary side inflorescence pairs | Kurgan area effect in the absence of isolation | *glmmTMB(Prim_side_inf_pairs ~ hload_sc + log_habitat_area_sc + date_number_sc + c_year + (1 \| site_id/trans_id/plot_id/plant_id) , data= sideinf2_large, REML= TRUE, control= glmmTMBControl(optCtrl= list(iter.max= 1e3,eval.max= 1e3)), ziformula=~1, family= compois(link = "log"))* | | *(Intercept)* | *0.040* | *0.151* | *<0.001* | *R^2^*c=0.285; *R^2^* = 0.137 | 0.719 |
|  |  |  |  | **Heat load** | **-0.229** | **0.082** | **0.008** |  |  |
|  |  |  |  | Kurgan area | 0.120 | 0.080 | 0.171 |  |  |
|  |  |  |  | **Date of visit** | **0.192** | **0.081** | **0.001** |  |  |
|  |  |  |  | **year 2022** | **-0.003** | **0.150** | **0.019** |  |  |
|  |  |  |  | **year 2023** | **0.340** | **0.148** |  |  |  |
|  | Isolation effect in the absence of kurgan area | *glmmTMB(Prim_side_inf_pairs ~ hload_sc + Hanski_sc + date_number_sc + c_year + (1 \| site_id/trans_id/plot_id/plant_id), data= sideinf2_large, REML= TRUE, control= glmmTMBControl(optCtrl= list(iter.max= 1e3,eval.max= 1e3)), ziformula=~1, family= compois(link = "log"))* | | *(Intercept)* | -0.005 | 0.205 | *<0.001* | *R^2^*c= 0.343; *R^2^*m= 0.141 | 0.719 |
|  |  |  |  | **Heat load** | **-0.259** | **0.117** | **0.014** |  |  |
|  |  |  |  | Hanski index | 0.134 | 0.141 | 0.275 |  |  |
|  |  |  |  | **Date of visit** | **0.292** | **0.094** | **<0.001** |  |  |
|  |  |  |  | **year 2022** | **-0.027** | **0.170** | **0.009** |  |  |
|  |  |  |  | **year 2023** | **0.458** | **0.189** |  |  |  |

**Table S4.** Details of models of *Salvia nemorosa* traits from which either area or Hanski index were removed, using data collected on 11 kurgans and two flat reference grasslands (“all sites” dataset). The first column shows the response variable, the second and third columns indicate the model structure, the fourth column indicates the explanatory variables. The next columns show the coefficient means (β) and standard errors SE (β), the p value determined by ANOVA tests, the conditional (*R^2^*c) and marginal (*R^2^*m) R squared values of the model and the p values corresponding to the Kolmogorov-Smirnov test (KS) of model residuals distribution. Significant effects are shown in bold letters.

| **Response variable** | **Model** | | **Model structure** | **Explanatory variables** | **Estimate (β)** | **SE (β)** | **p** | ***R^2^*** | **p (K.S.)** |
| --- | --- | --- | --- | --- | --- | --- | --- | --- | --- |
| Stem height | Kurgan area effect in the absence of isolation | *glmmTMB(log(stem_hei_noinf_allplants) ~ hload_sc + log_habitat_area_sc + c_year + (1 \| site_id/trans_id/plot_id/plant_id), data= stemhe_noNA1, REML= TRUE, control= glmmTMBControl(optCtrl= list(iter.max= 1e3,eval.max= 1e3)), family= gaussian(link= "identity"))* | | *(Intercept)* | *5.901* | *0.042* | *<0.001* | *R^2^*c = 0.770  *R^2^*m = 0.567 | 0.117 |
|  |  |  |  | **Heat load** | **-0.159** | **0.027** | **<0.001** |  |  |
|  |  |  |  | **Habitt area** | **-0.095** | **0.035** | **0.008** |  |  |
|  |  |  |  | **year 2022** | **-0.347** | **0.018** | **<0.001** |  |  |
|  |  |  |  | **year 2023** | **0.119** | **0.017** |  |  |  |
|  | Isolation effect in the absence of kurgan area | *glmmTMB(log(stem_hei_noinf_allplants) ~ hload_sc + Hanski_sc + c_year + (1 \| site_id/trans_id/plot_id/plant_id), data= stemhe_noNA1, REML= TRUE, control= glmmTMBControl(optCtrl= list(iter.max= 1e3,eval.max= 1e3)), family= gaussian(link= "identity"))* | | *(Intercept)* | *5.888* | *0.043* | *<0.001* | *R^2^*c = 0.774  *R^2^*m = 0.566 | 0.212 |
|  |  |  |  | **Heat load** | **-0.160** | **0.027** | **<0.001** |  |  |
|  |  |  |  | Hanski index | -0.077 | 0.039 | 0.060 |  |  |
|  |  |  |  | **year 2022** | **-0.346** | **0.018** | **<0.001** |  |  |
|  |  |  |  | **year 2023** | **0.120** | **0.017** |  |  |  |
| Mean leaf area | Kurgan area effect in the absence of isolation | *glmmTMB(log(mean_leafarea) ~ hload_sc + log_habitat_area_sc + c_year + (1 \| site_id/trans_id/plot_id/plant_id), data= leafarea_noNA1, REML= TRUE, control= glmmTMBControl(optCtrl= list(iter.max= 1e3,eval.max= 1e3)), family= gaussian(link= "identity"))* | | *(Intercept)* | *7.626* | *0.062* | *<0.001* | *R^2^*c = 0.467  *R^2^*m = 0.186 | 0.013 |
|  |  |  |  | **Heat load** | **-1.656** | **0.050** | **0.002** |  |  |
|  |  |  |  | Habitat area | 0.005 | 0.052 | 0.906 |  |  |
|  |  |  |  | **year 2022** | **-0.241** | **0.040** | **<0.001** |  |  |
|  |  |  |  | **year 2023** | **0.177** | **0.039** |  |  |  |
|  | Isolation effect in the absence of kurgan area | *glmmTMB(log(mean_leafarea) ~ hload_sc + Hanski_sc + c_year + (1 \| site_id/trans_id/plot_id/plant_id), data= leafarea_noNA1, REML= TRUE, control= glmmTMBControl(optCtrl= list(iter.max= 1e3,eval.max= 1e3)), family= gaussian(link= "identity"))* | | *(Intercept)* | *7.623* | *0.059* | *<0.001* | *R^2^*c = 0.480  *R^2^*m = 0.188 | 0.015 |
|  |  |  |  | **Heat load** | **-0.170** | **0.049** | **<0.001** |  |  |
|  |  |  |  | Hanski index | 0.040 | 0.053 | 0.398 |  |  |
|  |  |  |  | **year 2022** | **-0.238** | **0.040** | **<0.001** |  |  |
|  |  |  |  | **year 2023** | **0.180** | **0.038** |  |  |  |
| Number of stems | Kurgan area effect in the absence of isolation | *glmmTMB(num_stems) ~ hload_sc + log_habitat_area_sc + c_year + (1 \| site_id/trans_id/plant_id), data= numstems_noNA1, REML= TRUE, control= glmmTMBControl(optCtrl= list(iter.max= 1e3,eval.max= 1e3)), family= nbinom2(link = "log"))* | | *(Intercept)* | *1.937* | *0.091* | *<0.001* | *R^2^*c = 0.791  *R^2^*m = 0.050 | 0.197 |
|  |  |  |  | **Heat load** | **-0.134** | **0.062** | **0.034** |  |  |
|  |  |  |  | Habiat area | -0.083 | 0.077 | 0.236 |  |  |
|  |  |  |  | **year 2022** | **-0.131** | **0.047** | **<0.001** |  |  |
|  |  |  |  | **year 2023** | **-0.347** | **0.045** |  |  |  |
|  | Isolation effect in the absence of kurgan area | *glmmTMB(num_stems) ~ hload_sc + Hanski_sc + c_year + (1 \| site_id/trans_id/plot_id/plant_id), data= numstems_noNA1, REML= TRUE, control= glmmTMBControl(optCtrl= list(iter.max= 1e3,eval.max= 1e3)), family= nbinom2(link = "log"))* | | *(Intercept)* | *1.921* | *0.094* | *<0.001* | *R^2^*c = 0.792  *R^2^*m = 0.045 | 0.093 |
|  |  |  |  | **Heat load** | **-0.142** | **0.065** | **0.032** |  |  |
|  |  |  |  | Hanski index | -0.008 | 0.085 | 0.922 |  |  |
|  |  |  |  | **year 2022** | **-0.127** | **0.047** | **<0.001** |  |  |
|  |  |  |  | **year 2023** | **-0.344** | **0.045** |  |  |  |
| Inflorescence length | Kurgan area effect in the absence of isolation | *glmmTMB(inf_len ~ hload_sc + log_habitat_area_sc + date_number_sc + c_year + (1 \| site_id/trans_id/plot_id/plant_id) , data= inflen1_large, REML= TRUE, control= glmmTMBControl(optCtrl= list(iter.max= 1e3,eval.max= 1e3)), family= tweedie(link = "log"))* | | *(Intercept)* | *4.848* | *0.053* | *<0.001* | *R^2^*c = 0.423  *R^2^*m = 0.241 | 0.054 |
|  |  |  |  | Heat load | -0.017 | 0.034 | 0.663 |  |  |
|  |  |  |  | Habitat area | 0.046 | 0.043 | 0.239 |  |  |
|  |  |  |  | Date of visit | -0.002 | 0.024 | 0.860 |  |  |
|  |  |  |  | **year 2022** | **-0.470** | **0.048** | **<0.001** |  |  |
|  |  |  |  | **year 2023** | **-0.009** | **0.052** |  |  |  |
|  | Isolation effect in the absence of kurgan area | *glmmTMB(inf_len ~ hload_sc + Hanski_sc + date_number_sc + c_year + (1 \| site_id/trans_id/plot_id/plant_id), data= inflen1_large, REML= TRUE, control= glmmTMBControl(optCtrl= list(iter.max= 1e3,eval.max= 1e3)), family= tweedie(link = "log"))* | | *(Intercept)* | *4.854* | *0.056* | *<0.001* | *R^2^*c = 0.426  *R^2^*m = 0.237 | 0.131 |
|  |  |  |  | Heat load | -0.016 | 0.036 | 0.773 |  |  |
|  |  |  |  | Hanski index | 0.030 | 0.047 | 0.519 |  |  |
|  |  |  |  | Date of visit | -0.0002 | 0.024 | 0.202 |  |  |
|  |  |  |  | **year 2022** | **-0.471** | **0.048** | **<0.001** |  |  |
|  |  |  |  | **year 2023** | **-0.009** | **0.052** |  |  |  |
| Number of primary side inflorescence pairs | Kurgan area effect in the absence of isolation | *glmmTMB(prim_side_inf_pairs ~ hload_sc + log_habitat_area_sc + date_number_sc + c_year + (1 \| site_id/trans_id/plot_id/plant_id) , data= sideinf1_large, REML= TRUE, control= glmmTMBControl(optCtrl= list(iter.max= 1e3,eval.max= 1e3)), ziformula= ~1, family= compois(link = "log"))* | | *(Intercept)* | *-0.096* | *0.232* | *<0.001* | *R^2^*c = 0.408  *R^2^*m = 0.147 | 0.624 |
|  |  |  |  | **Heat load** | **-0.259** | **0.133** | **0.042** |  |  |
|  |  |  |  | Habitat area | -0.121 | 0.151 | 0.362 |  |  |
|  |  |  |  | **Date of visit** | **0.305** | **0.099** | **<0.001** |  |  |
|  |  |  |  | **year 2022** | **-0.079** | **0.186** | **0.018** |  |  |
|  |  |  |  | **year 2023** | **0.451** | **0.208** |  |  |  |
|  | Isolation effect in the absence of kurgan area | *glmmTMB(prim_side_inf_pairs ~ hload_sc + Hanski_sc + date_number_sc + c_year+ (1 \| site_id/trans_id/plot_id/plant_id) , data= sideinf1_large, REML= TRUE, control= glmmTMBControl(optCtrl= list(iter.max= 1e3,eval.max= 1e3)), ziformula= ~1, family= compois(link = "log"))* | | *(Intercept)* | *-0.127* | *0.236* | *<0.001* | *R^2^*c = 0.419  *R^2^*m = 0.148 | 0.848 |
|  |  |  |  | **Heat load** | **-0.276** | **0.136** | **0.033** |  |  |
|  |  |  |  | Hanski index | -0.041 | 0.162 | 0.818 |  |  |
|  |  |  |  | **Date of visit** | **0.306** | **0.099** | **0.001** |  |  |
|  |  |  |  | **year 2022** | **-0.059** | **0.186** | **0.018** |  |  |
|  |  |  |  | **year 2023** | **0.472** | **0.209** |  |  |  |

**Table S5.** Details of the model of *Salvia nemorosa* flowering probability. The first three columns show the response variable, the model structure, and the explanatory variables respectively. The next columns show the coefficient means (β) and standard errors SE (β), the p value determined by ANOVA tests, the conditional (*R^2^*c) and marginal (*R^2^*m) R squared values of the model and the p values corresponding to the Kolmogorov-Smirnov test (KS) of model residuals distribution. Significant effects are shown in bold letters.

| **Response variable** | **Model structure** | **Explanatory variables** | **Estimate (β)** | **SE (β)** | **p** | ***R^2^*** | **p (K.S.)** |
| --- | --- | --- | --- | --- | --- | --- | --- |
| Flowering state (yes or no) | *glmer(flowering ~ stem_hei_noinf_allplants_sc * c_year +*  *(1 \| site_id/trans_id/plot_id/plant_id), data= alldatflow, family= binomial(link= "logit"),*  *control= glmerControl(optimizer= "bobyqa", optCtrl= list(maxfun=2e5)))* | *(Intercept)* | *1.028* | *0.431* | *0.017* | *R^2^*c = 0.707  *R^2^*m = 0.478 | 0.534 |
|  |  | **Stem height** | **1.627** | **0.212** | **<0.001** |  |  |
|  |  | **year 2022** | **2.204** | **0.324** | **<0.001** |  |  |
|  |  | **year 2023** | **0.633** | **0.231** | **0.006** |  |  |
|  |  | **Stem height:year 2022** | **2.048** | **0.345** | **<0.001** |  |  |
|  |  | **Stem height:year 2023** | **0.643** | **0.238** | **0.007** |  |  |
